# Regulation mechanism of microRNAs in cardiac cells-derived exosomes in cell crosstalk

**DOI:** 10.3389/fphar.2024.1399850

**Published:** 2024-08-20

**Authors:** Shanshan Lin, Yuanjian Yang, Zhou Zhou, Wen Li, Xianliang Wang, Yu Liu, Yingfei Bi, Jingyuan Mao

**Affiliations:** ^1^ First Teaching Hospital of Tianjin University of Traditional Chinese Medicine, National Clinical Research Center for Chinese Medicine Acupuncture and Moxibustion, Tianjin, China; ^2^ Tianjin University of Traditional Chinese Medicine, Tianjin, China; ^3^ Jinling Hospital, Nanjing University School of Medicine, Nanjing, China

**Keywords:** cardiac cells-derived exosome, microRNA, cell crosstalk, mechanism research, review

## Abstract

The heart is a multicellular system, and the intercellular crosstalk mechanism is very important for the growth and development of the heart and even the organs, tissues, and cells at a distance. As a kind of extracellular vesicle, exosomes are released by different types of cells and can carry specific genetic material, endosomal proteins, cytokines, etc., which are the main material basis for mediating cell crosstalk mechanism. Among them, microRNA carried by cardiac cells-derived exosomes have highly conserved sequences and play a key role in regulating the function of organs, tissues, and cells related to cardiovascular diseases and their complications and comorbidities, which have attracted extensive attention in the medical community in recent years. Following up on the latest research progress at home and abroad, this review systematically summarized the regulatory role of microRNA from cardiac cells-derived exosomes in various cell crosstalk, including not only cardiac cells (including cardiomyocytes, fibroblasts, myofibroblast, cardiac progenitor cells, cardiac microvascular endothelial cells, cardiosphere-derived cells, etc.) but also tumor cells, bone marrow progenitor cells, and other tissue cells, in order to provide a reference for the prevention and treatment of cardiovascular diseases and their complications and comorbidities.

## 1 Background

As an important organ of the body, the heart can rely on its impulse conduction system and myocardial systolic function to pump blood, ensure adequate blood oxygen supply to organs and tissues of the whole body, and thus maintain the functional state of the body. There is a great diversity of cells in different regions of the heart, and each region has a specific subpopulation of cells (Litviňuková et al., 2020). In addition to cardiomyocytes (CMs), fibroblasts (FBs), and myofibroblasts (MFBs), which account for 80–90% of the total, a mature heart also includes potentially adult “stem cells” (cardiac progenitor cells (CPCs) and mesenchymal stem cells), endothelial cells (ECs), mesothelial cells, pericytes, smooth muscle cells, resident macrophages, immune system-relate cells, etc. These distinct cell subpopulations are not isolated from each other in the heart, but interact with each other to maintain the function of the whole organ. And the cross-talk mechanism between these cell subpopulations is important for the growth and development of cells, tissues, and organs (Tirziu et al., 2010).

Extracellular vesicle is a general term for all kinds of vesicles with membrane structure released by cells, which can stably carry some important signaling molecules from donor cells and participate in information exchange, substance exchange, and mechanism interaction between cells (Baranyai et al., 2015). Depending on their diameter and pathogenesis, extracellular vesicles can be further divided into subgroups of exosomes, microvesicles, and apoptotic bodies (Baranyai et al., 2015). Apoptotic corpuscles are released by apoptotic cells. The microvesicles are shed from the plasma membrane. Exosomes are secreted by most cell types via the kernel-poly vesicular complex. Among them, the important regulation mechanism mediated by exosomes has received extensive attention in the medical field in recent years. The International Extracellular Vesicle Society (ISEV) is the largest community of extracellular vesicle researchers in the world, founded in Sweden in September 2011. As a global advocate in the field of extracellular vesicle research, ISEV has been committed to advancing global extracellular vesicle research, effectively promoting the global extracellular vesicle research in basic, translational, and clinical directions. Exosomes are a class of extracellular vesicles with a diameter of about 30–150 nm and a lipid bilayer structure. The biogenesis of exosomes begins with the early stage of endosome formation from the inward budding of the cytoplasmic membrane. During this process, endosomes integrate membrane proteins, lipids, and soluble proteins associated with the extracellular environment into the cell. In addition, the trans-Golgi network and endoplasmic reticulum contribute to the formation of early endosomes (Kalluri and LeBleu, 2020). Endosomal membrane invagination produces many intraluminal vesicles, and late endosomes containing multiple intraluminal vesicles are called multivesular bodies (Piper and Katzmann, 2007). Some multivesular bodies can fuse with the plasma membrane and be released into the extracellular matrix as exosomes (Baranyai et al., 2015; Kalluri and LeBleu, 2020). Current studies have shown that exosomes are commonly found in different types of cells, and the released exosomes can be found in various body fluids such as blood, urine, ascites, pericardial fluid, synovial fluid, amniotic fluid, and breast milk (Xu et al., 2019). Exosomes can carry abundant specific genetic material (such as DNA, mRNA, microRNA (miRNA), IncRNA, circRNA, ribosomal RNA), endosomal proteins (such as tetra trans proteins (CD9, CD63, CD81), ALG-2 interacting protein X, tumor susceptibility gene 101, Clathrin, annexin A5), cytokines, chemokines, growth factors, lipids, polysaccharides, and cell metabolites from donor cells (Xu et al., 2019). These contents can be selectively released into the extracellular space in the form of exosomes under different pathophysiological conditions and are delivered functionally into recipient cells, which is an important material basis for exosomes to regulate cell function. Among them, miRNA is an endogenous, short (about 20–24 nucleotides in length), highly conserved single-stranded non-coding RNA, which is produced by Dicer enzyme processing of 70–90 base-sized single-stranded RNA precursors with hairpin structure. Mature miRNAs can be combined with other proteins to form an RNA-induced silencing complex, which can cause target mRNA degradation or translation inhibition, thereby regulating post-transcriptional gene expression and participating in various pathologic and physiological processes (McGeary et al., 2019). A single miRNA can target multiple genes, and multiple miRNAs can act on the same target, so miRNAs often participate in the “network” of interactions between cells (Inukai and Slack, 2013). Currently, the therapeutic potential of exosomal miRNAs as biomarkers for disease diagnosis and progression has been confirmed and strengthened.

**FIGURE 1:**
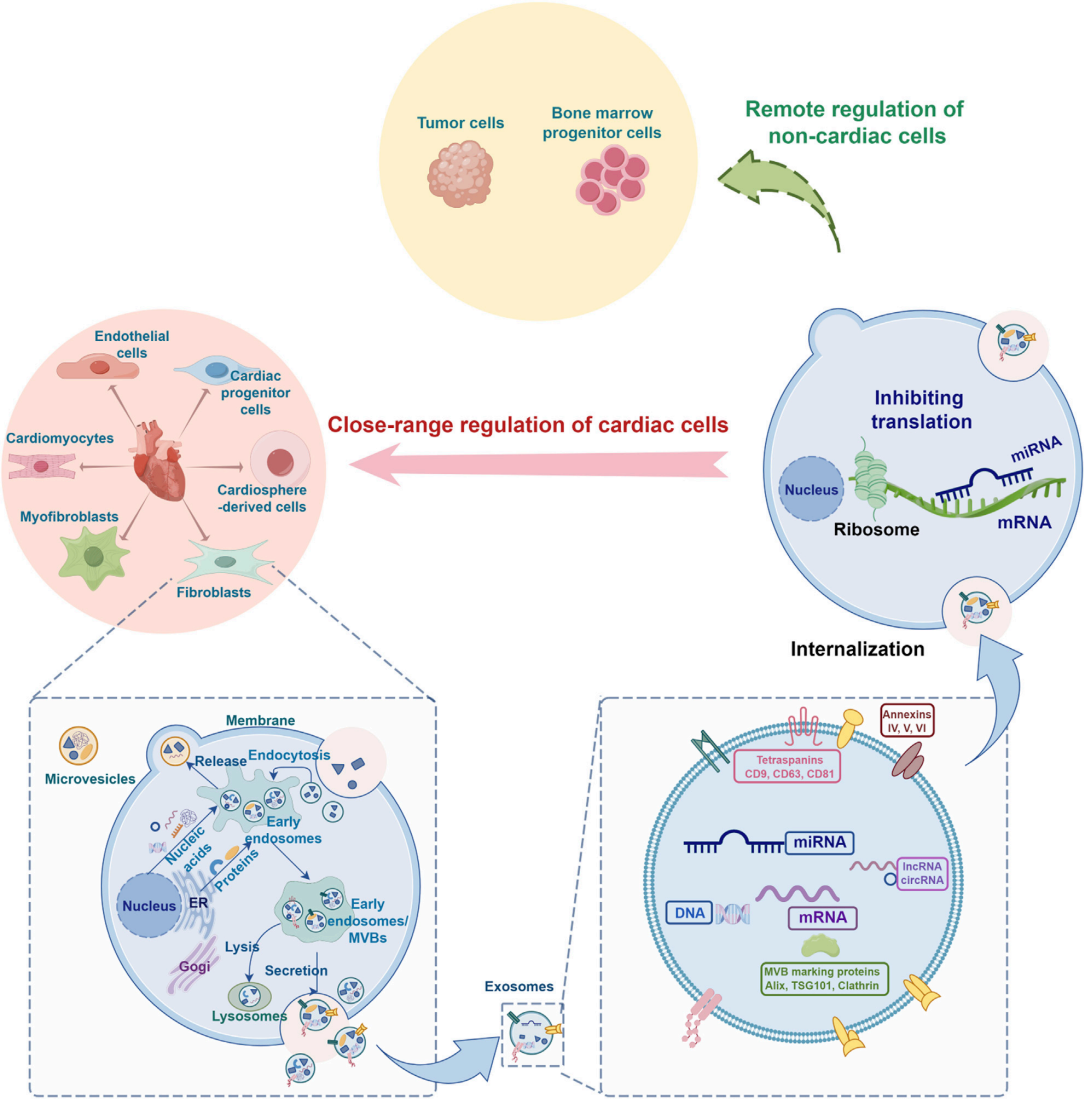
Schematic diagram of the regulatory role of microRNA from cardiac cells-derived exosomes in cell crosstalk (By FigDraw). ER, endoplasmic reticulum; MVB, multivesicular body; Alix, ALG-2 interacting protein X; TSG101, tumor susceptibility gene 101.

Cardiac cells-derived exosomes are the generic term for exosomes released by various heart cells. MiRNAs carried by cardiac cells-derived exosomes not only play an important role in the growth and differentiation of cells and the occurrence and development of diseases related to the cardiovascular system but also play an important role in trans-organ remote regulation (Figure 1). Following the latest research progress at home and abroad, this review systematically summarizes the regulatory role of miRNAs from cardiac cells-derived exosomes in various cell crosstalk, with a view to providing a new direction for the prevention and treatment of cardiovascular diseases and their complications and comorbidities.

## 2 Regulation of miRNAs from cardiac cells-derived exosomes on different cardiovascular cells

### 2.1 Studies on miRNAs in exosomes derived from CMs

As previously reported, researchers divided the heart into six regions (including the left atrium, right atrium, left ventricle, right ventricle, apex, and ventricular septum), and completed the human heart atlas study by using large-scale single-cell sequencing and nuclear transcriptome sequencing, combined with machine learning, fluorescence imaging, and other technical methods. The results of the atlas analysis showed that CMs accounted for the largest proportion of cells in the heart tissue, with 30.1% in the atrial region and 49.2% in the ventricular region (Litviňuková et al., 2020). CMs (also known as cardiac muscle cells or cardiac myocytes) are the muscle cells (myocytes) that make up the heart muscle. In addition to the general atrial and ventricular muscle working cells, the generalized CMs also include specially differentiated cardiac muscle cells, that is, the cardiac pacemaker cells that dominate the heart rhythm (composed of the sinus node, the atrial tract, the atrioventricular junction, the atrioventricular tract, and Purkinje fibers). The former are the true cardiac muscle cells that build up the muscle walls (called myocardium) of both atria (the chambers in which blood enters the heart) and the ventricles (the chambers where blood is collected and pumped out of the heart). These working cells do not have an automatic rhythm, but have excitability and the ability to get excited, and need the stimulation of external stimuli to generate excitement, that is, by shortening and lengthening muscle fibers to generate the pumping force of the heart, so that the heart muscle pumps blood throughout the body, and these electrical stimuli are spontaneously generated and conducted by the cardiac pacemaker cells. In other words, the main function of CMs is to generate and transmit excitation, which plays an important role in controlling the contraction, relaxation, and activity rhythm of the myocardium.

#### 2.1.1 Regulation of CMs by miRNAs in CMs-derived exosomes

Studies have shown that the expression level of miRNAs released by CMs-derived exosomes in pathological states can change to different degrees compared with normal conditions, and miRNAs with significantly different expressions can promote or alleviate the development of disease by regulating the function of CMs. At present, the potential of exosomal miRNAs to mediate cardiac repair or injury in a variety of cardiovascular diseases has been demonstrated. Huang et al. (2021) found that the level of miR-328-3p in CMs-derived exosomes of patients with myocardial infarction was significantly increased, and this exosome could promote CMs apoptosis by regulating the Caspase signaling pathway after uptake by normal CMs, leading to the deterioration of myocardial infarction. This proapoptotic process can be reversed by Z-DEVD-FMK, an inhibitor of caspase-3 (Huang et al., 2021). Zhang et al. (2020) found that exosomes rich in miR-30a can be highly enriched in the serum of patients with myocardial infarction and play a protective role in CMs. In addition, gallate can inhibit CMs apoptosis and autophagy induced by ischemia-reperfusion (I/R) by increasing the average diameter and concentration of CMs exosomes as well as the expression level of miR-30a mRNA and specific protein in exosomes, thereby reducing acute myocardial infarction (Zhang et al., 2020). Therefore, both miR-328-3p and miR-30a in exosomes are expected to be potential new diagnostic markers and therapeutic targets for myocardial infarction.

#### 2.1.2 Regulation of FBs by miRNAs in CMs-derived exosomes

MiRNAs in exosomes are key molecules mediating the communication between CMs and FBs. Cardiac fibrosis is mainly mediated by activated FBs (i.e., MFBs), and miRNAs in CMs-derived exosomes can be transferred to FBs through the cargo form of exosomes and promote their fibrosis process (Yang et al., 2018). CMs can release more exosomes after adverse stimulation such as hypoxia or angiotensin II, and the expression level of miR-208a in exosomes is significantly increased. After endocytosis by FBs, these miR-208A-rich exosomes inhibited the expression of dual specificity tyrosine phosphorylation regulated kinase 2 (DYRK2) in FBs and promoted dephosphorylation of activated nuclear factor of activated T cells in the cytoplasm, thus promoting the proliferation of FBs and transformation into MFBs (Yang et al., 2018). Hypoxia-induced CMs-derived exosomes with high expression of miR-208a/b were added to FBs, which significantly inhibited the apoptosis of FBs, promoted survival and migration, and enhanced the expression of α smooth muscle actin (α-SMA), type I collagen and type III collagen. In addition, it can further enhance the accumulation of reactive oxygen species, malonaldehyde, and Fe^2+^, which are major indicators of iron death, and inhibit the expression of glutathione peroxidase 4 (GPX4), a key regulator of iron death (Guo et al., 2023).

Cardiac stress overload can significantly increase the mechanical overtension of CMs and FBs in myocardial tissue, and then induce the activation of FBs through direct action and paracrine mechanism of signal transmission between cells in myocardial tissue, leading to myocardial fibrosis and cardiac dysfunction. It was found that miR-494-3p carried by CMs-derived exosomes could inhibit phosphatase and tensin homolog deleted on chromosome ten (PTEN) expresses and activates AKT/Smad2/3/ERK signaling pathway to regulate FBs activation, which is closely related to the occurrence and development of myocardial fibrosis induced by stress overload, and specific inhibition of miR-494-3p in myocardial tissue can significantly reverse this pathological process (Tang, 2020). *In vitro* and *in vivo* studies have shown that miR-217 in CMs-derived exosomes can also enhance the proliferation of FBs by directly targeting PTEN, and participate in the process of cardiac hypertrophy and fibrosis (Nie et al., 2018).

In addition, the activation of FBs is essential for healing after myocardial infarction. Morelli et al. (2019) found that miR-195 was significantly upregulated in CMs-derived exosomes of mice with myocardial infarction and could be transferred to FBs to participate in the activation of MFBs (Morelli et al., 2019), which could provide new ideas for the research and treatment strategies of cardiac repair after myocardial infarction.

#### 2.1.3 Regulation of ECs by miRNAs in CMs-derived exosomes

ECs are the type of noncardiomyocyte that occupies the largest proportion of the heart (Pinto et al., 2016), and can be divided into endocardial ECs, cardiac microvascular endothelial cells (CMECs), and coronary ECs according to their distribution locations. Among them, CMECs are distributed in capillaries throughout the myocardium and are connected to each other through circulation, forming a continuous endothelium between CMs. In the local oxidative stress environment after myocardial infarction, apoptosis, necrosis, proliferation and migration of CMECs are limited, which seriously damages the integrity of microvessels and leads to inadequate perfusion of CMs after coronary artery recirculation, which further leads to apoptosis or necrosis of originally viable CMs and changes in cardiac structure and function under various pathophysiological effects. These lesions eventually lead to cardiac insufficiency and cardiac death. When pressure overload occurs, CM hypertrophy occurs and interstitial component deposition increases, resulting in cardiac hypertrophy. In hypertrophic myocardial tissue, microvascular content is relatively insufficient, which leads to relative ischemia and hypoxia in these tissues, and the continuous progression of the disease will lead to heart failure (Garcia et al., 2015; Garcia et al., 2016). Therefore, the close anatomical relationship between CMs and ECs is essential for maintaining vascular homeostasis and cardiac function.

Ischemia and hypoxia are the main pathological basis of CM dysfunction. Ischemic CMs-derived exosomes can promote the proliferation and germination of ECs, stimulate the formation of capillary-like structures, enhance adhesion complexes and barrier properties, and their cardioprotective effects may be related to differentially expressed miRNAs. Gou et al. (2020) found that the expression level of miR-19a-3p in the serum of patients with myocardial infarction was significantly increased. Further *in vitro* studies have confirmed that overexpressed miR-19a-3p can inhibit the proliferation and angiogenesis of ECs by down-regulating hypoxic inducible factor-1 α (HIF-1α) by targeting exosomes in CMs treated with H_2_O_2_. Wang et al. (2014) found that miR-320 expression in CMs-derived exosomes in Goto-Kakizaki rats (a commonly used animal model of type 2 diabetes) was significantly increased compared with CMs-derived exosomes in adult Wistar rats. Effective uptake of miR-320-enriched exosomes by ECs can inhibit ECs proliferation, migration, and tubular formation, while knockdown of miR-320 can reverse exosome-mediated angiogenesis inhibition in Goto-Kakizaki rats. In addition, Ottaviani LM et al. conducted cell experiments and found that miR-200c-3p was highly enriched in the exosomes released by CMs after pathological stimulation with phenylephrine and isoproterenol. Direct transfection of ECs with miR-200c-3p precursor molecules or co-culture of stimulated CMs with ECs via transwell also inhibited the ability of ECs to proliferate, migrate, and form tubes, and this effect could be eliminated by exosome inhibitors. Ottaviani et al. (2019) conducted further animal experiments and found that inhibiting the expression of miR-200c-3p could reduce myocardial hypertrophy in mice with cardiac stress overload induced by transverse aortic constriction, reduce the fibrotic region, increase the number of capillaries, and preserve cardiac ejection fraction

#### 2.1.4 Regulation of Gli1+ cells by miRNAs in CMs-derived exosomes

Gli^1+^ cells are vascular smooth muscle cell progenitors that differentiate into highly differentiated smooth muscle cells in acute vascular injury but differentiate into osteoblast-like cells and lead to vascular calcification in chronic vascular injury. Gli1^+^ cells can promote the formation of regenerative H blood vessels, which can be used for tissue regeneration and repair. It was found that hypoxic-treated CMs-derived exosomes promoted significantly up-regulated levels of fibrosis-related protein α-SMA, collagen receptor-discoid domain receptor 2, and collagen I in Gli1^+^ cells, thus promoting the conversion of Gli1^+^ cells to fibrotic phenotype, which may be related to the high expression of miR-223 in exosomes (Lin, et al., 2019).

#### 2.1.5 Regulation of macrophage by miRNAs in CMs-derived exosomes

Yu et al. (2021) found that miR-155 plays a crucial role in the initiation of macrophage inflammation, and angiotensin II-induced hypertrophic CMs derived exosomes can induce the phosphorylation of extracellular regulated kinase (ERK), c-Jun N-terminal kinase (JNK), and p38 through the enrichment of miR-155, thus stimulating the inflammatory response of macrophages. In addition, the expression level of miR-130 is also significantly increased in exosomes derived from atrial myocytes treated with angiotensin II, and exosomes can promote M1-type polarization of macrophages by transferring miR-130 (Huang et al., 2022). M1-type polarization of macrophages is also promoted by the enrichment of miR-106b-5p in exosomes released by CMs induced by oxidative stress (Li et al., 2023).

### 2.2 Studies on miRNAs in exosomes derived from FBs

FBs are the most common cells in the connective tissue of the heart and are differentiated from embryonic mesenchymal cells. These cells have strong functional activities and large cell bodies, which can synthesize and secrete collagen fibers, elastic fibers, reticular fibers, and organic substrates. Fibrosis is characterized by excessive proliferation and activation of MFBs as the main components, resulting in a large amount of collagen and other extracellular matrix deposition, as well as the destruction of muscle tissue structure and function decline. Activation of FBs in local tissues is the main source of MFBs. In damaged tissues, some FBs exist in the form of their activated state, myofibroblast, which participates in tissue repair and promotes the fibrotic process of the injured area by synthesizing extracellular matrix (Hinz et al., 2007; Zheng et al., 2014; Men et al., 2017).

#### 2.2.1 Regulation of CMs by miRNAs in FBs-derived exosomes

In the acute stage of myocardial I/R injury, the expression of fibroblast exosome miR-423-3p is significantly upregulated, which can enhance cell viability and reduce cell apoptosis by inhibiting the expression of RAS-related protein RAP2C in H9c2 cells, thus playing a cardioprotective role. Ischaemic post-conditioning (Postcon) can enhance this effect by up-regulating the expression of miR-423-3p in fibroblast exosomes/microvesicles (Luo et al., 2019). GATA binding protein 4 (GATA4) is an early heart-specific transcription factor. Overexpression of GATA4 can enhance the protective effect of exosomes derived from cardiac colony-forming unit fibroblasts (cCFU-FBs) on myocardial ischemic injury. The mechanism is partly attributed to the fact that miR221 in GATA4 overexpressed cCFU-FBs-derived exosomes can inhibit the expression of PTEN and activate the PTEN/PI3K/AKT signaling pathway, thereby alleviating the apoptosis of CMs (Hao et al., 2020). miR-133a is a muscle-specific miRNA, which can be specifically expressed in cardiac muscle and skeletal muscle and is involved in cardiac development and pathophysiological processes. Under the condition of myocardial I/R injury, the expression of miR-133a in fibroblast exosomes was also significantly upregulated. After exosomes are taken up by CMs, miR-133a inhibits the pyroptosis of CMs by targeting ELAV-like RNA binding protein 1 (ELAVL1), thereby protecting CMs (Liu et al., 2022). However, other *in vitro* and *in vivo* studies have shown that miR-143 in exosomes derived from FBs may promote CMs apoptosis (Zhang, 2018), suggesting that FBs may play a bidirectional regulatory role in myocardial injury. In addition, proteomic analysis showed that the exosomes secreted by FBs could significantly enrich miR-21-3p. miR-21-3p is a potent paracrine signaling medium that may induce CMs hypertrophy by inhibiting its target sorbin binding SH3 domain protein 2 (SORBS2) and PDZ binding LIM domain 5 (PDLIM5) (Bang et al., 2014).

#### 2.2.2 Regulation of ECs by miRNAs in FBs-derived exosomes

Transforming growth factor-β1 (TGF-β1) is one of the most effective pro-fibrotic factors, which can significantly activate FBs and promote collagen type 1 α1 chain (COL1α1), periostin (POSTN), and fibronectin (FN1) gene expression and phosphorylation of Smad2/3 and p38. The level of miR-200a-3p in FBs-derived exosomes treated with TGF-β1 is significantly increased and can act on ECs, inhibiting the proliferation, migration, and tubular formation of ECs and promoting apoptosis by down-regulating the expression of vascular endothelial growth factor A (VEGFA), HIF-1α, CD31, and angiopoietin 1 gene (Ranjan et al., 2021).

### 2.3 Studies on miRNAs in exosomes derived from MFBs

The transformation of FBs to MFBs is a key event in the pathological mechanism of fibrosis, and atrial fibrosis is one of the common pathological processes of atrial fibrillation. Angiotensin II was found to induce the transformation of FBs into MFBs, and α-SMA expression was significantly upregulated. At the same time, miR-21-3p can be enriched in exosomes derived from MFBs, and its action on CMs can lead to the downregulation of the expression of L-type calcium channel Cav1.2, a marker of ionic remodeling associated with atrial fibrillation in CMs, which may be a key substance in increasing the susceptibility to atrial fibrillation in the process of atrial fibrosis (Li et al., 2020).

### 2.4 Studies on miRNAs in exosomes derived from CPCs

Progenitor cells function between stem cells and adult cells. Compared with stem cells that can differentiate into various types of cells, progenitor cells are more certain in the direction of differentiation, and usually can only differentiate into specific cells of the tissue in which they are located, and are the “reserve army” of tissues and organs. CPCs are a group of mesenchymal stem cells derived from myocardial tissue with certain differentiation abilities. Pedigree tracing studies have found that CPCs cannot directly regenerate CMs after myocardial injury, but the paracrine function and tubular formation ability of these CPCs are very important for the repair of infarcted myocardes (Gao et al., 2021).

#### 2.4.1 Regulation of CPCs by miRNAs in CPCs-derived exosomes

Studies have shown that oxidative stress causes CPCs to secrete more exosomes. Compared with normal CPCs-derived exosomes, the expression of miR-133a was significantly enhanced in CPCs-derived exosomes induced by oxidative stress, and overexpressed miR-133a could be transported from exosomes to recipient cells to protect CPCs from cell death by down-regulating the expressions of pro-apoptotic genes Bim and Bmf (Izarra et al., 2014).

#### 2.4.2 Regulation of CMs by miRNAs in CPCs-derived exosomes

Compared with exosomes released by normal CPCs, miR-21 was significantly upregulated in exosomes released by CPCs induced by oxidative stress. However, the same oxidative stress resulted in low expression of miR-21 and high expression of cleaved caspase-3 in H9c2 cells. The programmed cell death 4 (PDCD4) gene is the target gene of miR-21. H9c2 pretreated by CPCs-derived exosomes can exhibit significant upregulation of miR-21, and overexpressed miR-21 in exosomes can counteract oxidative stress-induced apoptosis by down-regulating PDCD4 (Xiao et al., 2016).

Barile et al. (2014) found that miRNAs in CPCs-derived exosomes could inhibit the apoptosis of mouse HL-1 cells and promote the tubular formation of human umbilical vein endothelial cells (HUVECs). Compared with FBs, the most enriched miRNA in the exosomes released by CPCs included miR-210, miR-132, and miR-146a-3p. MiR-210 is an important hypoxia-associated miRNA, which has cardioprotective effects such as inhibiting CMs apoptosis, promoting angiogenesis, and improving cardiac function, and may be a potential biomarker for predicting or diagnosing cardiovascular diseases. miR-210 inhibited CMs apoptosis by down-regulating its target ephrin A3 and PTP1b, while miR-132 enhanced tubular formation in ECs by down-regulating its target RasGAP-p120. Cervio et al. (2014) found that transfection of HL-1 cells with pre-miR-323-5p, pre-miR-181a, and pre-miR-132 significantly enhanced cell tolerance to hypoxia/reoxygenation (H/R) injury, while hypoxia further increased the expression of cellular protective miRNAs in CPCs-derived exosomes.

Endogenous GATA4-positive (GATA4pos) cells play a key role in cardiac protection after myocardial injury. *In vitro* experiments showed that the expression of miR-222 in the isolated and purified GATA4pos CPCs-derived exosomes was significantly increased compared with the unsorted CPCs-derived exosomes. This exosome can protect H9c2 cells from apoptosis and improve their survival rate by mediating the PTEN-PI3K/AKT signaling pathway (Yu et al., 2015). After being taken up by H9c2 cells, the highly miR-451 enriched exosomes released by CPCs can also protect H9c2 cells from oxidative stress damage by inhibiting the activation of caspase-3/caspase-7 (Chen et al., 2013). In addition, *in vitro* experiments have shown that miR-528-3p and miR-7641 are the most effective miRNAs to induce the proliferation of static CMs, among which miR-7641 plays a role by inhibiting Hippo signal transduction pathway (increasing the YAP/pYAP ratio) in part (Sharma et al., 2018).

The clinical drugs doxorubicin and trastuzumab both have certain cardiotoxicity, which may be related to the upregulation of two known miR-146a-5p target genes Traf6 and Mpo (signaling mediators encoding the axis of inflammation and cell death) in CMs. In doxorubicin-treated CPCs-derived exosomes, miR-146a-5p is highly enriched and can reduce oxidative stress damage induced by doxorubicin or trastuzumab by inhibiting the expression of Traf6 and Mpo (Milano et al., 2020).

#### 2.4.3 Regulation of ECs by miRNAs in CPCs-derived exosomes

Hypoxia-induced CPCs-derived exosomes can enhance the tubular formation of ECs (Gray et al., 2015). Compared with exosomes derived from normal human dermal fibroblasts (NHDFBs), multiple miRNAs were enriched in exosomes derived from CPCs. They included miR-210 and miR-146a, which inhibited CMs apoptosis through downregulating the ephrinA3/PTP1b signaling pathway and Nox4 target protein respectively, and miR-132, which promoted angiogenesis through downregulating RasGAP-p120 target protein, etc (Barile et al., 2015). After 12 h treatment with exosomes released by hypoxic CPCs, miR-103 and miR-15b in ECs also showed an increasing trend. In addition, other studies have used exosomes derived from bioengineered CPCs transfected with pro-angiogenic miR-322 to culture human ECs, resulting in a more pronounced angiogenic response (Youn et al., 2019), providing a new direction for the treatment of ischemic cardiovascular diseases.

### 2.5 Studies on miRNAs in exosomes derived from CMECs

The bioactive substances produced and secreted by CMECs play an important role in maintaining vascular tension, regulating blood pressure, and anti-thrombosis. *In vitro* studies have shown that miR-27b-3p is highly enriched in hypoxic-induced CMECs-derived exosomes. Compared with normal culture conditions, CMECs-derived exosomes showed more obvious improvement in H/R-induced H9c2 cell damage under hypoxia conditions. The expression of pyroptosis-related factors GSDMD, NLRP3, and caspase-1 in H9c2 cells could be downregulated by regulating Foxo1/GSDMD axis, thereby inhibiting pyroptosis. At the same time, serum creatine kinase isozyme, lactate dehydrogenase, interleukin-1β, and interleukin-18 levels were significantly reduced (Zhang, 2023).

### 2.6 Studies on miRNAs in exosomes derived from cardiosphere-derived cells (CDCs)

Cardiospheres (CSP) are interstitial cells derived from atrial or ventricular tissue. After adherent growth, myocardial tissue is then planted in a polylysine-coated Petri dish to form a heterogeneous cell combination with a three-dimensional structure, which is named CSP because it could be suspended and cultured to form 3D spherical cell clusters (Messina et al., 2004). CDCs are monolayer adherent cells formed after CSP is digested and cultured in a culture dish coated with adhesion protein (Smith et al., 2007). They have the characteristics of stem cells and can differentiate into CMs and vascular ECs *in vitro*. A number of studies have suggested that CDCs have certain therapeutic effects on infarcted myocardia, and it has been found that the beneficial effects of CDCs-derived exosomes on injured myocardia may be mediated by miRNA.

#### 2.6.1 Regulation of CMs by miRNAs in CDCs-derived exosomes

*In vitro* studies showed that CDCs-derived exosomes could significantly inhibit apoptosis and promote cell proliferation of CMs. *In vivo* studies have shown that CDCs-derived exosomes contribute to functional recovery after myocardial infarction in mice (Messina et al., 2004). MiRNA microarray analysis showed that, compared with NHDFBs, many miRNAs in CDCs-derived exosomes were significantly upregulated, including miR-146a, miR-22, miR-24, and miR-210, etc., which could partially explain the myocardial protective effect of CDCs exosomes (Ibrahim et al., 2012; Ibrahim et al., 2013).

#### 2.6.2 Regulation of ECs by miRNAs in CDCs-derived exosomes

Under hypoxia-induced conditions, the levels of pro-angiogenic miRNA, including miR-126, miR-130a, and miR-210, in CDCs-derived exosomes were significantly increased compared with those under normal oxygen conditions. CDCs-derived exosomes cultured under hypoxic conditions at a concentration of 25 μg/mL can promote the tubular formation of HUVECs by enriching miRNAs in the exosomes that promote angiogenesis, thus playing a significant benefit in the treatment of heart disease (Namazi et al., 2018).

#### 2.6.3 Regulation of macrophage by miRNAs in CDCs-derived exosomes

Animal experiments have shown that compared with exosomes released by inert fibroblasts (Fb_exo_), CDCs-derived exosomes can reduce the infarct size, reduce the number of macrophages in infarct tissues and change the polarization of macrophages in myocardial infarction reperfusion model animals. Further studies showed that CDCs-derived exosomes significantly enriched miR-146a, miR-181b and miR-126 compared with Fbexo, among which miR-181b is an important candidate medium for CDCs-induced macrophage polarization. Protein kinase Cδ is a downstream target of miR-181b. CDCs-derived exosomes can play a cardioprotective role by transferring miR-181b to macrophages and reducing the level of protein kinase Cδ transcript (de Couto et al., 2017).

## 3 Regulation of distant cells by miRNAs in cardiac cells-derived exosomes

The contents of exosomes are released in the adjacent microenvironment, but some can also act on distant cells, tissues, or organs. Based on this, miRNAs derived from cardiac cells can not only participate in the proliferation, differentiation, and regeneration of heart-related cells but also transfer directly to distant organs and recognize resident cells after being transported by exosomes, mediating the crosstalk mechanism between heart cells and distant cells (Pu et al., 2021). However, there are relatively few studies on the crosstalk mechanism between heart cells and distant cells mediated by miRNAs in exosomes derived from heart cells.

### 3.1 Regulation of tumor cells by miRNAs in cardiac cells-derived exosomes

Iron death is a widespread form of non-apoptotic cell death that is expected to develop into a new strategy for cancer treatment. Yuan et al. (2023) found that the expression level of miR-22-3p was significantly upregulated in both CMs-derived exosomes from mice with chronic myocardial infarction and plasma-derived exosomes from patients with heart failure. *In vitro* studies have confirmed that overexpressed miR-22-3p in exosomes can directly target the acyl-CoA synthetase long-chain family member 4 (gene name: ACSL4) gene of tumor cells, thereby inhibiting cell iron death induced by iron death inducers of Ras and ST, which is one of the important mechanisms of accelerating tumor progression in heart failure.

### 3.2 Regulation of bone marrow progenitor cells by miRNAs in cardiac cells-derived exosomes

The expression of many miRNAs is tissue-specific, and the miRNAs specifically expressed in muscle are named the myomiRs family, including miR-1, miR-133a, miR-206, miR208a, miR-208b, miR-499a, miR-486, and so on. Cheng et al. (2019) proposed that myocardial miRNAs in circulating blood are carried by exosomes and mediate functional crosstalk between the ischemic heart and bone marrow. Myocardial infarction was associated with increased expression of myocardial miRNAs in circulating blood, and exosomes mediated the transfer of miR-1, miR-208, and miR-499 to bone marrow mononuclear cells. The uptake of exosomes enriched with myocardial miRNAs by receptor cells inhibits the expression of C-X-C motif chemokine receptor 4 (CXCR4) and indirectly increases the content of circulating progenitor cells, which may be an important mechanism by which myocardial exosomes carry miRNAs into the circulatory system to regulate the systemic response induced by heart injury (Cheng et al., 2019).

At present, there are relatively few studies on the mechanism of crosstalk between heart cells and distant cells mediated by miRNAs in cardiac cells-derived exosomes. The above findings provide some evidence support for the hypothesis that miRNAs in cardiac cells-derived exosomes can regulate the mechanism of crosstalk between heart cells and distant cells. It is helpful to explore new treatment strategies for cardiovascular disease complications and comorbidities.

Table 1 systematically summarizes the regulatory role of miRNAs from cardiac cells-derived exosomes in various cell crosstalk. The types and action pathways of miRNAs involved in previous studies are very complex and there is little overlap, suggesting that the current relevant studies are far from enough, and a broader and complex unknown world in this field needs to be explored.

**TABLE 1:** Regulatory role of miRNAs from cardiac cells-derived exosomes in various cell crosstalk.

Donor cells	Recipient cells	Enriched miRNA	Regulatory role	References
CMs after MI	Normal CMs	miR-328-3p	miR-328-3p regulates the Caspase signaling pathway and promotes the apoptosis of normal CMs.	Huang et al. (2021)
CMs treated with epigallocatechin gallate	I/R-induced CMs	miR-30a	miR-30a inhibits CMs apoptosis and autophagy induced by I/R by increasing the average diameter and concentration of CMs exosomes as well as the expression level of miR-30a mRNA and specific protein in exosomes.	Zhang et al. (2020)
Fibrotic CMs	Normal FBs	miR-208a	miR-208a inhibits the expression of DYRK2 in FBs, promotes the dephosphorylation of activated T nuclear factor in the cytoplasm, and then promotes the proliferation of FBs and the transformation into myoblasts.	Yang et al. (2018)
Hypoxic CMs	FBs	miR-208a/b	miR-208a/b inhibits the apoptosis of FBs, promotes survival and migration, enhances the accumulation of α-SMA, type I collagen, type III collagen, reactive oxygen species, malonaldehyde, and Fe^2+^, and inhibits the expression of GPX4, a key regulator of iron death.	Guo et al. (2023)
Hypertrophic CMs stimulated by mechanical overstretch for 24 h	FBs	miR-494-3p	miR-494-3p regulates FBs activation by inhibiting PTEN expression and activating the AKT/Smad2/3/ERK signaling pathway and is closely related to the occurrence and development of myocardial fibrosis caused by stress overloading.	Tang (2020)
H9c2 transfected with miR-217 mimics	FBs	miR-217	miR-217 targets PTEN to enhance the proliferation of FBs and participate in cardiac hypertrophy and fibrosis.	Nie et al. (2018)
CMs after MI	FBs after MI	miR-195	miR-195 is significantly upregulated in CMs-derived exosomes and can be transferred to FBs to participate in MFBs activation.	Morelli et al. (2019)
CMs treated with H_2_O_2_ for 3 h	ECs treated with H_2_O_2_ for 3 h	miR-19a-3p	miR-19a-3p inhibits ECs proliferation and tubular formation by down-regulating HIF-1α protein levels, and induces, cell death.	Gou et al. (2020)
CMs from Goto-Kakizaki rats	ECs	miR-320	miR-320 inhibits ECs proliferation, migration, and tubular formation.	Wang et al. (2014)
CMs stimulated by phenylephrine and isoproterenol	ECs	miR-200c-3p	miR-200c-3p inhibits ECs proliferation, migration and tubule formation.	Ottaviani et al. (2019)
Hypoxic CMs	Gli1^+^ cells	miR-223	miR-223 significantly up-regulates the levels of Gli1^+^ fibrosis-associated protein α-SMA, collagen receptor-discoid domain receptor 2, and type I collagen.	Lin et al. (2019)
Angiotensin II-induced CMs	Macrophages	miR-155	miR-155 induces ERK, JNK, and p38 through the enrichment of miR-155, thereby stimulating the inflammatory response of macrophages.	Yu et al. (2021)
Atrial muscle cells treated with angiotensin II	Macrophages	miR-130	miR-130 promotes M1-type polarization of macrophages.	Huang et al. (2022)
Oxidative stress-induced CMs	Macrophages	miR-106b-5p	miR-106b-5p promotes M1-type polarization of macrophages.	Li et al. (2023)
CMs induced by oxidative stress	Hypoxic H9c2	miR-423-3p	miR-423-3p promotes M1-type polarization of macrophages.	Luo et al. (2019)
cCFU-FBs overexpressed by GATA4 gene	H9c2	miR-221	miR-221 inhibits the expression of PTEN and activates the PTEN/PI3K/AKT signaling pathway, thereby alleviating the apoptosis of CMs.	Hao et al. (2020)
I/R injury-induced FBs	CMs	miR-133a	miR-133a targets ELAVL1 to inhibit the pyrodeath of CMs, thereby protecting CMs.	Liu et al. (2022)
Fibroblasts subjected to mechanical stretch for 48 h	CMs	miR-143	miR-143 promotes apoptosis of CMs.	Zhang (2018)
FBs	CMs	miR-21-3p	miR-21-3p inhibits CMs targets SORBS2 and PDLIM5 and induce CMs hypertrophy.	Bang et al. (2014)
FBs treated with TGF-β1	CMs	miR-200a-3p	miR-200a-3p inhibits the expression of VEGFA, HIF-1α, CD31, and angiopoietin 1 gene, inhibits proliferation, migration, and tubular formation, and promotes apoptosis.	Ranjan et al. (2021)
Atrial MFBs induced by angiotensin II	CMs	miR-21-3p	miR-21-3p down-regulates the expression of L-type calcium channel Cav1.2, a marker of ionic remodeling associated with atrial fibrillation in CMs, which may be a key substance in increasing the susceptibility to atrial fibrillation in the process of atrial fibrosis.	Li et al. (2020)
Sca-1^+^ CPCs after MI	CPCs induced by H_2_O_2_	miR-133a	miR-133a promotes CPCs survival under oxidative stress by decreasing Caspase-3 activity and targeting the expression of pro-apoptotic genes Bim and Bmf.	Izarra et al. (2014)
CPCs induced by oxidative stress	H9c2	miR-21	miR-21 down-regulates PDCD4 and inhibits oxidative stress-induced apoptosis of H9c2 cells.	Xiao et al. (2016)
CPCs	CMs and ECs	miR-210 and miR-132	miR-210 inhibited CMs apoptosis by down-regulating its target ephrin A3 and PTP1b; miR-132 enhanced tubular formation in ECs by down-regulating its target RasGAP-p120.	Barile et al. (2014)
Sca-1^+^ CPCs	HL-1 cells	pre-miR-323-5p, pre-miR-181a, and pre-miR-132	Enhance cell tolerance to H/R damage.	Cervio et al. (2014)
Endogenous GATA4 positive CPCs	H9c2	miR-222	miR-222 protects H9c2 cells from apoptosis by mediating the PTEN-PI3K/AKT signalling pathway and improves their survival rate.	Yu et al. (2015)
CPCs	H9c2 treated with H_2_O_2_ for 4 h	miR-451	miR-451 protects H9c2 cells from oxidative stress by inhibiting the activation of Caspase-3/Caspase-7.	Chen et al. (2013)
CPCs	iPSCs-derived CMs or CMs from newborn rats	miR-528-3p and miR-7641	miR-528-3p and miR-7641 induced the proliferation of stationary CMs.	Sharma et al. (2018)
CPCs treated with doxorubicin	CMs induced by doxorubicin or trastuzumab	miR-146a-5p	miR-146a-5p attenuates oxidative stress damage induced by adriamycin/trastuzumab by inhibiting the expression of Traf6 and Mpo.	Milano et al. (2020)
CPCs	CMs and ECs	miR-210, miR-146a and miR-132	miR-210 and miR-146a inhibited apoptosis of CMs by down-regulating ephrinA3/PTP1b signaling pathway and Nox4 target protein. miR-132 plays a pro-angiogenic role by down-regulating the target protein RasGAP-p120.	Barile et al. (2015)
Bioengineered CPCs transfected by pro-angiogenic miR-322	ECs	miR-322	miR-322 promotes angiogenesis.	Youn et al. (2019)
Hypoxic induced CMECs	H/R-induced H9c2	miR-27b-3p	miR-27b-3p down-regulates the expression of GSDMD, NLRP3 and Caspase-1 in H9c2 cells by regulating Foxo1/GSDMD axis, thereby inhibiting pyroptosis. At the same time, the serum creatine kinase isozyme, lactate dehydrogenase, interleukin-1β, and interleukin-18 levels were significantly reduced.	Zhang (2023)
CDCs	H_2_O_2_-induced CMs	miR-146a, miR-22, miR-24, miR-210, etc.	miR-146a, miR-22, miR-24, miR-210, etc. inhibited CMs apoptosis and promoted cell proliferation.	Ibrahim et al. (2013)
Hypoxic CDCs	HUVECs	miR-126, miR-130a, miR-210, and other pro-angiogenic miRNAs	miR-126, miR-130a, miR-210, and other pro-angiogenic miRNAs promote tubular formation of HUVECs.	Namazi et al. (2018)
CDCs	Macrophages after ischemic preconditioning	miR-181b	miR-181b is an important candidate mediator for CDCs-induced macrophage polarization and plays a cardioprotective role by reducing PKCδ transcription levels.	de Couto et al. (2017)
CMs after MI	Tumor cells	miR-22-3p	miR-22-3p directly targets the ACSL4 gene of tumor cells, thereby inhibiting cell iron death induced by the eradicator of Ras and ST and accelerating tumor progression.	Yuan et al. (2023)
CMs after MI	Bone marrow progenitor cells	miR-1, miR-208, and miR-499	miR-1, miR-208, and miR-499 inhibited CXCR4 expression and increased the number of CPCs.	Cheng et al. (2019)

α-SMA, α smooth muscle actin; ACSL4, acyl-CoA synthetase long chain family member 4; AMI, acute myocardial infarction; cCFU-FBs, cardiac colony-forming unit fibroblasts; CMECs, cardiac microvascular endothelial cells; CMs, cardiomyocytes; CPCs, cardiac progenitor cells; CXCR4, C-X-C motif chemokine receptor 4; DYRK2, dual specificity tyrosine phosphorylation regulated kinase 2; ECs, endothelial cells; ELAVL1, ELAV-like RNA binding protein 1; ERK, extracellular regulated kinase; FBs, fibroblasts; GPX4: glutathione peroxidase 4; H/R, hypoxia/reoxygenation; HIF-1α, hypoxic inducible factor-1 α; UVECs, human umbilical vein endothelial cells; I/R; ischemia-reperfusion; iPSCs, induced pluripotent stem cells; JNK, c-Jun N-terminal kinase; MFBs, myofibroblasts; MI, myocardial infarction; miRNA, microRNA; PDCD4: programmed cell death 4; PTEN, tensin homolog deleted on chromosome ten; TGF-β1, transforming growth factor-β1; VEGFA, vascular endothelial growth factor A.

## 4 Discussion and prospect

The heart is one of the main organs to maintain the life activities of the body and can achieve sufficient blood oxygen supply for all organs and tissues of the body. With the progression of heart disease, pathological changes in different heart cells occur one after another and interact with each other. In addition, abnormal changes in the function and structure of the heart are often accompanied by dysfunction of other organs and tissues, and the treatment of heart disease can also play a certain intervention role in the complications and complications of heart disease, and these effects mainly depend on the crosstalk mechanism between different cells. Cell crosstalk refers to the interaction between different cell types, which may occur at any stage of cell physiological and pathological processes, including cell proliferation, differentiation, apoptosis, and cell function changes. Although each cell has its own relatively independent signal transduction system, which seems to have no influence on each other, in fact, various signal pathways of different cells often need to be intertwined to form an information network to work together.

Evidence has increasingly shown that exosomes are the key mediators in the regulation of different cell crosstalk mechanisms. During disease progression, miRNAs can be released into the peripheral circulatory system by diseased tissues in the form of exosomes, and disease-related circulating miRNA levels change significantly as a result. MiRNAs are protected by phospholipid bilayer in exosomes, which effectively avoids the process of inactivation and degradation, and ensures the stability of its expression level to a certain extent. MiRNAs can mediate the long-distance communication between cells and participate in the regulation of physiological functions of various tissues, organs, and systems in the body. In addition, circulating miRNAs have the characteristics of high stability, sequence conservation and non-invasive detection, so miRNAs in exosomes have great potential to be biomarkers for the diagnosis and efficacy evaluation of various diseases. But at the same time there are some problems: The content of exosomes in body fluids is small, and the separation and purification process is still difficult. Neither the centrifugal method nor commercial kits can specifically completely separate exosomes. Exosomes isolated and purified from the medium still contain a large number of non-exosome components, such as functional vesicles such as microvesicles and apoptotic bodies, resulting in low purity of exosomes. It may affect the accuracy and reliability of exosomes in clinical applications. It is also important to note that the regulatory role of miRNAs in exosomes depends on the amount of exosomes released and the exosome’s tendency to specific heart cell types. Previous studies have shown that the release and uptake of exosomes from various cells may be affected by many factors, such as comorbidities and drugs, diet, exercise, lifestyle habits, and aging. Lark and LaRocca, (2022) examined the age-dependent expression (age coefficient) of genes involved in exosome biogenesis (22 genes), exosome cargo (3 genes), and senescence (5 genes). Of the 131 cell populations studied from different tissues, 95 had at least one exosome biogenetic gene affected by age, suggesting that the expression of exosome biogenetic genes is affected by age in many cell populations (Lark and Larocca, 2022). It can be seen that exosomal miRNAs face many difficulties in clinical application. In view of this, more accurate, standardized, rapid, and specific isolation and purification methods and body fluid biopsy techniques still need to be explored and clinically promoted to achieve the quality standardization of exosomal miRNAs.

Cardiac cells-derived exosomes are not morphologically different from other cell-derived exosomes, and cell origin and function are the main points of identification. Cardiac cells-derived exosomes are the generic term for exosomes secreted by various heart cells containing a large number of bioactive molecules. They are an important means of cardiac cell communication, which can not only regulate the function of neighboring heart cells, but also remotely regulate other cell types. Some of these special cardiac cells-derived exosomes can also be used as early diagnostic markers and therapeutic targets for heart disease. Since the current relevant studies are basically carried out at the cellular level, it is not necessary to identify the source and function of exosomes. This review focused on miRNAs in cardiac cells-derived exosomes, followed up on the latest research progress at home and abroad, and systematically summarized the regulatory mechanisms of miRNAs in various cardiac cells-derived exosomes in different cell crosstalk. MiRNAs in cardiac cells-derived exosomes can not only regulate cardiac cells (including CMs, FBs, ECs, CPCs, CDCs, smooth muscle cell progenitors, mesenchymal stem cells, macrophages, etc.) but also remotely regulate the life activities of tumor cells, bone marrow-derived progenitors and other cells across organs. However, the exact mechanism by which exosome derived miRNAs interact with recipient cells has not been fully elucidated.

Lionetti et al. (2014) performed a transmural (i.e., sub-epicardial, mesocardial and sub-endocardial layers) analysis of the left ventricular septum and antero-lateral free wall, and found that myocardial hibernation was present in the left ventricular region of failing hearts in both ischemic cardiomyopathy (ICM) and idiopathic dilated cardiomyopathy (DCM) patients undergoing cardiac transplantation. Compared with normal heart, left ventricular glycogen content was increased in DCM and ICM as compared with normal heart (*P* < 0.001). Capillary density was homogenously reduced in both DCM and ICM as compared with normal heart (*P* < 0.05), with a lower decrease independent of the extent of fibrosis in sub-endocardial and sub-epicardial layers of DCM as compared with ICM. These results suggest that the phenotype of a failing heart is regionally altered, and the regulation of miRNAs in cardiac cells-derived exosomes on different cells may be related to the regional phenotypic changes, but not to the pathogenesis. In addition, due to the fact that current relevant studies mainly observe the regulatory role of exosome miRNAs secreted by various disease model cells, while there are few studies on exosome miRNAs secreted by normal cells, a complete evidence system has not yet been formed. With the continuous development of research technology, it is important to carry out scientific, rigorous, economical and feasible research methods to further develop and expand the fields related to the transport, delivery and uptake of miRNAs in exosomes. In the future, with the in-depth development of relevant studies and the generation of research conclusions, we will further distinguish and summarize the functions of miRNAs from various cardiac cell-derived exosomes in different physiological and pathological states.

At present, the clinical application of miRNAs from cardiac cells-derived exosomes is still in the stage of gradual exploration, but it is believed that with the continuous development of miRNA chip high-throughput sequencing, nano-drug carriers, and other technologies, in-depth exploration of related studies will provide more possibilities for targeted therapy and drug carrier development and clinical application of heart diseases and their complications and complications.
